# Exploring Knowledge and Consumption of Beans Among Adults with Gluten-Related Disorders—A Cross Sectional Study in Canada

**DOI:** 10.3390/nu18213553

**Published:** 2026-10-28

**Authors:** Alicia Stahl, Alyssa Chapleau, Sana Tasawar, Safa Shahid, Niels J. Maywald, Nanette Stroebele-Benschop, Iris Joye, Dalia El Khoury

**Affiliations:** 1 Department of Family Relations and Applied Nutrition, University of Guelph, Guelph, ON N1G 2W1, Canada; 2 Department of Human Health Sciences, University of Guelph, Guelph, ON N1G 2W1, Canada; 3 Department of Nutritional Crop Physiology, University of Hohenheim, Fruwirthstraße 20, 70599 Stuttgart, Germany; niels.maywald@uni-hohenheim.de; 4 Department of Applied Nutritional Psychology, University of Hohenheim, Fruwirthstraße 12, 70599 Stuttgart, Germany; 5 Department of Food Science, University of Guelph, Guelph, ON N1G 2W1, Canada

**Keywords:** celiac disease, non-celiac gluten sensitivity, gluten-free diet, beans, consumption, barriers, knowledge, cross-sectional study

## Abstract

Introduction: Gluten-related disorders have been increasingly reported across all gender and age groups. In addition to their higher cost, gluten-free products are often higher in fat, sugar, and sodium. Despite the substantial nutritional benefits of beans, they remain insufficiently represented in gluten-free dietary patterns. Aim: In this study, our aim was to explore knowledge and consumption of beans among adults with gluten-related disorders, including celiac disease (CD) and non-celiac gluten sensitivity (NCGS). Methods: This study followed a cross-sectional, observational design. In collaboration with Celiac Canada, individuals with a diagnosis of CD or NCGS were recruited. Participants were asked to complete a Qualtrics-based questionnaire, exploring self-reported Diagnosis and Health Status, Eating Habits and Consumption of Beans, Knowledge, Determinants of Bean Consumption, Barriers and Drivers of Bean Consumption, and Demographics. Descriptive statistics as well as exploratory analyses and regression analyses were conducted, with statistical significance defined as *p* ≤ 0.05. Results: A total of 528 participants, including 490 individuals with CD and 38 with NCGS, completed the survey. Several self-reported micronutrient deficiencies were mentioned at the time of diagnosis, with iron-deficiency anemia being the most prevalent (69.0%). In addition, the most frequently self-reported symptom was fatigue (72.2%), followed by flatulence and abdominal pain (71.2%). Most participants (96.4%) strongly agreed that following a gluten-free diet is essential for the management of their diagnosis. Although a high proportion of participants (88.6%) reported daily consumption of plant-based foods, only 0.9% of participants reported daily consumption of dry beans and 0.4% reported daily consumption of canned beans. The most frequently mentioned motivators were the nutritional value of beans (74.9%), whereas the most common barrier to bean consumption was the long cooking and soaking time (40.2%). In addition, potential cross-contamination with gluten was mentioned as another major concern to including beans in gluten-free diets. Despite participants’ confidence in knowledge about beans and their nutritional value and health benefits, most participants (68.9%) agreed that knowing more about how to prepare beans would encourage them to consume them as part of their diets. Based on multivariable linear regression analysis, confidence in knowledge about bean preparation (*p* ≤ 0.001) and interest in learning more about them (*p* ≤ 0.001) were positively associated with increased bean consumption. Conclusions: Despite high interest in beans and strong confidence in bean-related knowledge among individuals with CD or NCGS, consumption remains relatively low. Educational resources focused on preparation, recipes, and gastrointestinal tolerance may help increase consumption. Future research should evaluate the tolerability and long-term health effects of bean consumption in CD and NCGS populations.

## 1. Introduction

Gluten-related disorders are a group of disorders characterized by immune-mediated responses to gluten protein, consisting of gliadin and glutenin subunits [1]. The disorder has been characterized into three types: autoimmune, allergic, and non-autoimmune/non-allergic [2]. Celiac disease (CD) is the most common autoimmune disease, predisposed by genetics, while gluten ataxia and dermatitis herpetiform are alternative clinical manifestations of CD [2]. The prevalence of CD in the global population ranges from 0.5 to 2% [3]. CD affects all genders and age groups, although women are 1.5 times more likely to be diagnosed, with 70% occurring after 20 years of age [4,5]. In contrast, gluten sensitivity is classified as non-immune/non-allergic gluten disorder where there is a biological reaction to gluten, however not mediated by the immune system [2]. An estimated worldwide occurrence of gluten sensitivity is 6.3% [6]. Gluten-related disorders are associated with both intestinal and extraintestinal symptoms, as well as a wide range of comorbidities, including autoimmune conditions, neurological disorders, certain cancers, and macro- and micronutrient deficiencies [7]. These disorders share many clinical features, such as bloating, abdominal pain, diarrhea, headaches, general fatigue, brain fog, muscle aches and low mood, making differential diagnosis particularly challenging [8,9]. In Canada, CD affects approximately 1% of the population, while the prevalence of non-celiac gluten sensitivity (NCGS) remains less clearly defined due to limited diagnostic standardization [3,10].

Despite the number of people with gluten-related disorders, the only treatment includes adopting a gluten-free diet (GFD). Although CD and NCGS differ in their clinical characteristics and underlying pathophysiology, both conditions require adherence to a GFD. Research shows that gluten-free products are 150–500% more expensive than regular foods in Canada [11]. The higher costs reflect the expenses associated with specialized ingredients, import fees, and the reliance on smaller-scale production facilities [11]. This increased financial burden highlights the need to incorporate inexpensive, naturally gluten-free foods into the diet. One example would be the inclusion of beans. Integrating beans into GFDs can enhance their nutritional density as beans are rich in essential nutrients such as protein and fibre [12]. Without appropriately selected gluten-free alternatives, the nutrients typically supplied through wheat fortification may not be adequately replaced, thereby increasing the risk of deficiencies [12]. A study found that individuals following a traditional GFD characterized by low nutrient density may be at risk of inadequate intake of carbohydrates, protein, calcium, iron, and fibre [12]. Gluten free products are often higher in fat, sugar, and sodium, as manufacturers modify these foods to compensate for the less desirable sensory attributes that can result from the removal of gluten [13]. As a result, many micronutrients such as iron, folate, calcium, selenium, magnesium, zinc, niacin, thiamine and riboflavin, as well as vitamins A and D are frequently insufficient, increasing the risk of nutritional deficiencies [13].

Although there are substantial nutritional benefits for individuals adhering to a GFD, beans remain insufficiently represented in dietary patterns, especially among those with gluten-related disorders. Some common barriers to consuming beans include a lack of knowledge about how to prepare them, gastrointestinal discomfort, general dislike and the overall sensory attributes of beans [14]. Bean consumption in young Canadian adults when compared against Hispanic immigrants is low [15]. An online survey for university students found that 34.17% of participants lacked knowledge about bean preparation [14]. In a Canadian Food Survey Study, commonly reported barriers to bean consumption included challenges with preparation and cooking, disliking beans, issues with taste and/or texture, flatulence or abdominal discomfort, lack of cultural familiarity, reluctance to try new foods, friends do not eat them and difficulty incorporating beans into meals [16,17]. However, these findings primarily reflect research conducted in the general population. It remains unclear whether the same barriers apply to individuals who require a GFD because of gluten-related disorders. In particular, evidence regarding bean-consumption patterns and knowledge about beans in these populations remains limited.

To address this gap in the literature, this study aimed to explore knowledge and consumption of beans among adults with gluten-related disorders, including CD and NCGS. Findings from this study may guide dietary modifications to the GFD to enhance its nutritional quality and overall nutrient density.

## 2. Materials and Methods

### 2.1. Participants and Study Design

This study followed a cross-sectional, observational design. Data were collected using an online-based Qualtrics survey. Recruitment was conducted in collaboration with Celiac Canada (CCA), a national charitable organization that supports people living with CD. To ensure privacy, all participants completed the survey anonymously. The study protocol was reviewed by the Research Ethics Board of the University of Guelph for compliance with federal guidelines for research involving human participants (REB#1559).

Inclusion criteria for this study included: (1) must be 18 years of age or older; (2) must be fluent in English; and (3) must have a self-reported diagnosis of CD or NCGS. The sample size calculation was executed using G*Power (version 3.1.9.7) with the following settings: F-tests, linear multiple regression, a priori, effect size f^2^ = 0.15, α error = 0.05, power = 0.95. This calculation was based on multivariable linear regression using composite scores from the questionnaire as the outcome variables. This analysis indicated a minimum required sample size of 138 participants. This sample size ensured sufficient statistical power to reliably detect true effects and reduce the likelihood of Type II error. Questionnaire-based studies of pulse and bean consumption have reported numbers of participants ranging from 89 to 505 [18,19]. Therefore, the minimum target of 138 participants fell well within the range described in the literature.

### 2.2. Development of the Questionnaire

A draft of the questionnaire was developed based on comprehensive research of the literature to date. A preliminary version of the questionnaire was developed and evaluated by experts for review and pretesting. The questionnaire was distributed to 8 experts in the field of CD, gastroenterology, and cereal and pulse research, as well as 20 graduate students from the Department of Family Relations and Applied Nutrition at the University of Guelph. Experts were affiliated with the University of Hohenheim, the University of Guelph, the University of Toronto and Mount Saint Vincent University. Reviewers were asked to evaluate the questionnaire based on brevity, clarity, length, number of items, repetition, language, response options, order, scale, bias, and the presence of double-barreled questions. A total of five revisions were submitted for the evaluation of the questionnaire. The final versions included modifications to wording, question order, formatting, and adjusting response options. The modified and final questionnaire consisted of 6 sections: Diagnosis and Health Status, Eating Habits and Consumption of Beans, Knowledge, Determinants of Bean Consumption, Barriers and Drivers of Bean Consumption, and Demographics. The format included a combination of multiple-choice questions, Likert-based scale responses, and open-ended responses. The first section, Diagnosis and Health, included four questions assessing self-reported diagnosis type, age at diagnosis, self-reported symptoms, and macronutrient deficiencies related to CD or NCGS. The questions were adapted from Stroebele-Benschop et al., who carried out a survey assessing life challenges and quality of life among people with CD [20]. The Eating Habits and Consumption of Beans section consisted of eight questions surrounding GFD and consumption patterns. These questions were adapted from a questionnaire developed by the team [14], with additional questions about the types of meals that include beans and eating locations. These questions were adapted from Doma et al. and Monge et al., who investigated perceptions and reasons for legume consumption [16,19]. The Knowledge section included eight questions assessing knowledge on fibre and protein intake from beans, health benefits, and preparation methods of beans. This section was adapted from a questionnaire previously developed by the team [14]. The fourth section, Determinants of Bean Consumption, included a rating-scale question assessing perceptions of beans and a question on interest in beans. The Barriers and Drivers of Bean Consumption section included two questions exploring factors that encourage or limit bean consumption. Response options were adapted from previous questionnaires examining motivators and barriers to bean and pulse consumption [16,17,18,19,21]. The last section, Demographics, collected data on gender, age, ethnicity, province of residence, educational qualification, and household income. The entire questionnaire was estimated to require approximately 20 min to complete.

### 2.3. Statistical Analysis

Data visualization and statistical analyses were performed using R (version 4.3.2). Descriptive statistics were summarized as counts and percentages and presented using tables and bar charts. As unanswered questions could not be reliably distinguished from intentional non-selection, percentages were calculated among participants reporting at least one symptom or nutritional deficiency, as well as among those reporting at least one motivator or barrier to bean consumption. Word clouds were generated from responses to the open-ended question regarding interest in learning more about beans, including preferred topics and delivery formats. Responses were categorized by topic and format, quantified, and log-transformed to improve readability. Consumption frequencies were coded from 1 (“I never eat beans”) to 6 (“On a daily basis”). The two scores were summed to generate an overall bean-consumption frequency score ranging from 2 to 12. The total scores were categorized as low (2–4), moderate (5–6), or high (7–12) bean consumption. Relative bean consumption (%) was calculated as [(overall bean consumption frequency score − 2)/10] × 100.

Reported motivators and barriers were coded as binary indicators based on participants’ selections from the respective multiple-response questions. The numbers of reported motivators and barriers were calculated by summing the selected items within each question. Participants with no recorded selections across an entire motivator or barrier question were treated as having missing data for the corresponding count because an unanswered question could not be reliably distinguished from an intentional selection of no items. Although the overall bean consumption score was discrete and bounded, multivariable linear regression was used to estimate adjusted differences in mean scores. Model assumptions were examined, and ordinal regression was conducted as a sensitivity analysis for Model B. Two complementary multivariable linear regression models were fitted to examine associations with overall bean consumption. The objective was primarily explanatory. Model A included 20 individual motivator and barrier indicators. One barrier (unwillingness to try new foods) selected by only two participants was excluded because of its very low prevalence. Model B included the numbers of reported motivators and barriers as separate predictors, together with interest in learning more about beans, five self-reported confidences in bean-related knowledge, and reported frequencies of animal- and plant-based-product consumption. These additional variables were included to examine whether the associations of motivator and barrier variables with overall bean consumption persisted after accounting for related interest, confidence in bean-related knowledge, and dietary habits. Both models adjusted for age, gender, education, annual household income, and self-reported diagnosis (CD or NCGS), selected on conceptual grounds because of their potential associations with food choices and bean consumption. For Model B, the two sparsely represented education categories of no diploma and secondary school diploma were combined into a single category (“High school diploma or less”) to improve estimation stability. The original education categories were retained in Model A.

Regression coefficients were reported as unstandardized estimates (B), with 95% confidence intervals and *p*-values calculated using heteroskedasticity-consistent HC3 standard errors. Multicollinearity, heteroskedasticity, and potentially influential observations were assessed using variance inflation factors (including degrees-of-freedom-adjusted generalized variance inflation factors for categorical predictors), the Breusch–Pagan test, and Cook’s distance and influence diagnostics, respectively. Model diagnostics indicated no substantial multicollinearity among predictors (maximum adjusted GVIF: 1.225 for Model A and 1.704 for Model B). Breusch–Pagan tests indicated heteroskedasticity in both models (both *p* < 0.002), supporting the use of HC3 standard errors. To account for multiple testing of the 20 individual motivator and barrier indicators in Model A, *p*-values were adjusted using the Benjamini–Hochberg false discovery rate procedure. Associations involving individual indicators were interpreted as exploratory. Primary regression analyses used complete cases. In Model B, 24 observations exceeded the Cook’s distance threshold of 4/*n*. Excluding these observations and one additional observation with leverage equal to one yielded a sensitivity sample of 390 participants, with similar associations for motivator count, barrier count, interest in learning more about beans, and self-reported confidence in knowledge of bean preparation. The primary models retained their original complete-case samples. Ordinal regression sensitivity analysis of Model B used seven ordered consumption categories (scores 2–7 and ≥8). Associations were directionally consistent with the linear model, although the Brant test suggested possible departures from the proportional-odds assumption (χ^2^ = 145.37, *df* = 110, *p* = 0.010). Findings were therefore interpreted cautiously.

Furthermore, exploratory comparisons of bean consumption were conducted outside the multivariable linear regression models. Kruskal–Wallis tests followed by Dunn’s post hoc comparisons with Bonferroni correction were used to assess differences across levels of interest in learning more about beans and groups reporting more motivators than barriers, equal numbers, or more barriers than motivators. Mann–Whitney U tests compared consumption scores according to whether the motivator nutritional value was reported as a motivator or gastrointestinal discomfort as a barrier. Statistical tests were two-sided, with *p* < 0.05 used as the nominal significance threshold. Given the cross-sectional study design, regression findings were interpreted as associations rather than causal effects.

## 3. Results

### 3.1. Participant Characteristics

**Figure 1:**
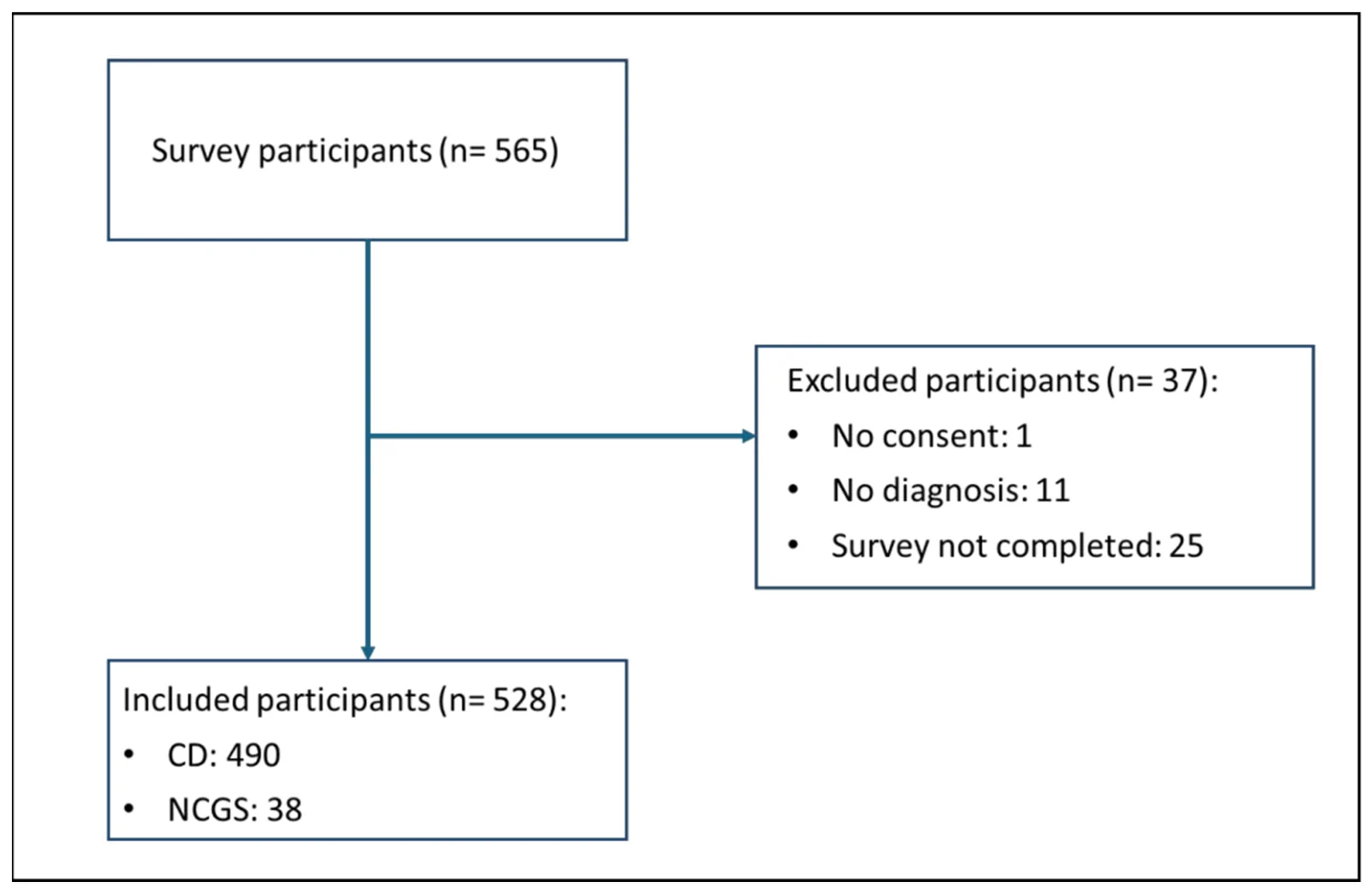
Participant flow diagram showing the number of individuals recruited, excluded, and included in the final analysis.

A total of 565 individuals participated in the study. Of these, 37 were excluded: 1 individual did not provide informed consent, 11 did not report a diagnosis of CD or NCGS, and 25 discontinued the survey prior to completion. The final analytical sample comprised 528 participants, including 490 individuals with CD and 38 with NCGS (Figure 1).

Participant demographics are presented in Table 1. The sample was composed of 91.3% females, 7.6% males, and 0.6% identifying as third gender. Ages ranged from 18 to 91 years, with the largest age groups being 36–45 years (22.7%) and 56–65 years (20.4%). The majority of participants identified as European/White (91.4%), while 2.4% reported Indigenous heritage. In terms of education, a large portion of participants held a bachelor’s degree (33.0%) or a postgraduate degree (30.9%). Over half of participants (53.5%) reported a household income of “$100,000 or more”, with smaller proportions represented in lower income categories. Most participants resided in Ontario (50.2%), followed by British Columbia (15.1%) and Alberta (13.0%). A smaller number of participants were distributed across other Canadian provinces and territories, with few residing outside of Canada.

### 3.2. Diagnosis of Participants

**Figure 2:**
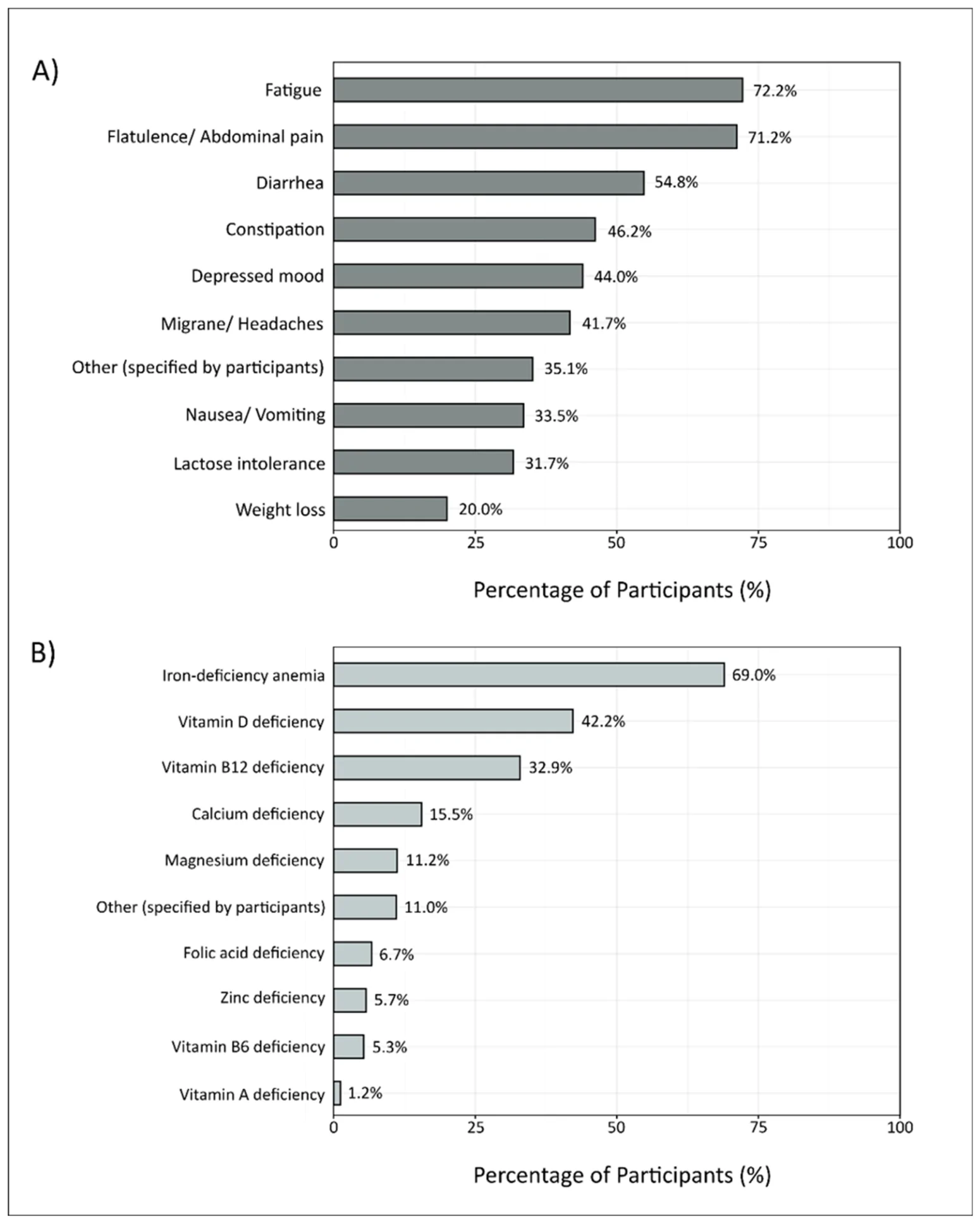
(**A**) Self-reported symptoms and intolerances occurring in conjunction with CD or NCGS. Percentages are based on responses from 496 participants who indicated at least one symptom. (**B**) Self-reported micronutrient deficiencies identified at the time of diagnosis of CD or NCGS. Percentages are based on responses from 419 participants who reported at least one deficiency. Multiple responses were allowed.

Participants varied in terms of age of diagnosis and nature of diagnosis. Most participants were diagnosed with CD (92.8%), while a smaller proportion were diagnosed with NCGS (7.2%). The age at diagnosis varied widely across the study population, ranging from childhood to adulthood. Most participants were diagnosed during adulthood, particularly between the ages of 26 and 64 years. Fewer participants were diagnosed earlier in life: 3.8% during childhood (0–11 years), 5.1% during adolescence (12–17 years), and 10.8% during young adulthood (18–25 years). Participants reported a range of self-reported symptoms commonly associated with either CD or NCGS. The most frequently reported symptom was fatigue (72.2%), followed by flatulence and abdominal pain (71.2%). Other commonly reported symptoms included diarrhea (54.8%) and constipation (46.2%). Additionally, several self-reported micronutrient deficiencies were mentioned at the time of diagnosis, with iron-deficiency anemia being the most prevalent (69.0%), followed by vitamin D deficiency (42.3%) and vitamin B12 deficiency (32.9%) (Figure 2).

### 3.3. Adherence to the Gluten-Free Diet

Dietary behaviours, adherence to, and perceived importance of the GFD were key aspects assessed among participants. Most participants (96.4%) strongly agreed that following a GFD is an important part of their daily lives, while a small proportion reported lower levels of agreement. Participants’ dietary intake patterns across various food categories were also evaluated. Most participants (79.7%) reported consuming animal-based products daily, while 1.1% consumed them rarely, and 0.8% reported never consuming these foods. Animal-based products included meat, fish, eggs, and dairy. Similarly, a high proportion of participants (88.6%) reported daily consumption of plant-based foods. A small proportion consumed these foods rarely (0.6%) or weekly (0.8%). Plant-based foods were defined as fruits and vegetables, whole grain cereals, legumes, and nuts. Participants’ consumption of beans, specifically dry and canned beans, was further assessed in detail in the following section.

### 3.4. Consumption Patterns of Beans and Bean-Containing Products

Participants’ bean consumption was assessed by asking how frequently they consume both dry and canned beans. Dry beans refer to beans that have been dried and are sold in sealed bags. They are uncooked and require soaking to prepare them prior to cooking. In contrast, canned beans are pre-cooked and commercially available in tin cans or glass jars. These beans are packed in a liquid that may contain salt, sugar or other preservatives. Participants reported that 30.1% never consume dry beans, while 6.1% indicated that they never eat canned beans and 23.9% reported that they rarely eat canned beans. Daily consumption of dry beans was reported by 0.9% of participants, 1.9% consumed them 4–5 times per week, and 16.3% consumed them 2–3 times per week. For canned beans, 0.4% reported daily consumption, 0.9% ate them 4–5 times per week, and 16.1% 2–3 times per week. Most participants rarely eat dry beans (45.1%) and consume canned beans once per week or less (52.7%). The frequency of consumption was based on both dry and canned bean intake. Overall, 39.8% of participants showed a low level of bean consumption, 47.9% showed a moderate level, and 12.3% showed a high level.

The patterns of bean consumption among participants were analyzed with a focus on typical meal occasions and preferred places of consumption. Many participants (87.8%) indicated dinner as their typical meal for eating beans, followed by lunch at 40.6% and breakfast at 5.4%. Some participants reported eating beans not only at breakfast, lunch, and dinner but also as snacks or desserts. The majority of participants reported eating beans mostly at home (63.4%), while a smaller proportion reported eating beans only at home (33.4%), mostly outside (6.1%) or only outside the home (1.4%). The survey also explored where participants prefer to eat beans when not at home. Restaurants (74.0%) were the most popular choice, followed by eating at someone else’s house (32.0%), cafeterias (5.0%) and kiosks (2.9%).

**Figure 3:**
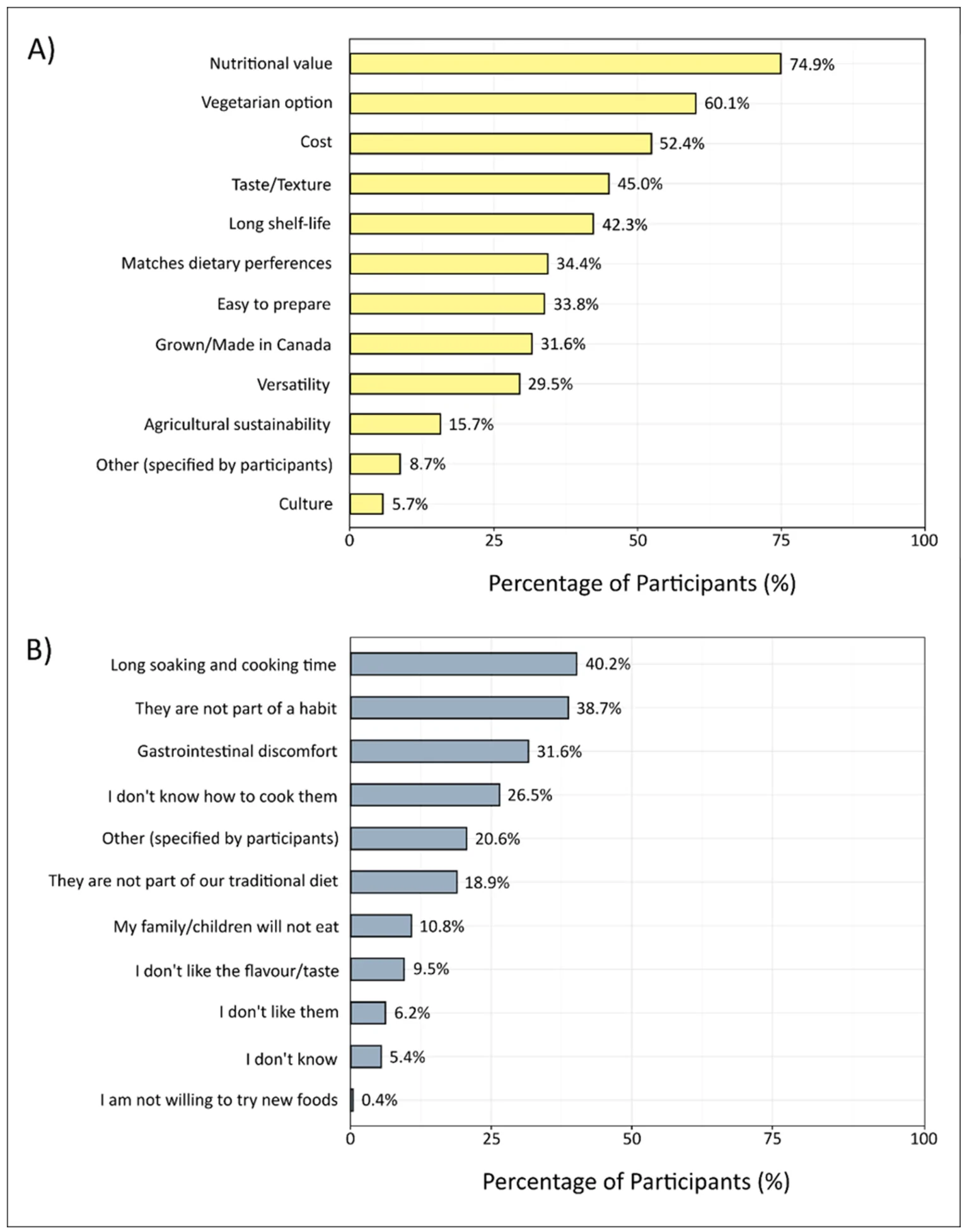
(**A**) Motivators for bean consumption. Percentages are based on responses from 471 participants who indicated at least one motivator. (**B**) Barriers to bean consumption. Percentages are based on responses from 465 participants who indicated at least one barrier.

Motivators and barriers to bean consumption for CD or NCGS were examined and reported in Figure 3. The most frequently mentioned motivators for bean consumption were its nutritional value (74.9%), their status as a vegetarian option (60.1%), the cost (52.4%), and the taste and texture of beans (45.0%). In contrast, the most common barriers to bean consumption were the long cooking and soaking time (40.2%), eating beans not being part of a habit (38.7%), gastrointestinal discomfort associated with bean consumption (31.6%) and a lack of knowledge about how to cook beans (26.5%). In addition, several participants expressed concerns about potential cross-contamination with gluten, which was mentioned as an additional reason to avoid beans. Based on the reported motivators and barriers, 315 participants reported more motivators than barriers, 116 reported an equal number of motivators and barriers, and 97 reported more barriers than motivators. Additionally, participants were asked whether learning about the health benefits of beans and bean-containing products would influence their consumption. Responses showed that 51.7% of participants agreed that knowing more about the health benefits of beans would increase their consumption, with 18.0% strongly agreeing and 33.7% somewhat agreeing. Participants were also asked if knowing more about ways to consume beans, such as recipes, cooking methods, or nutritional information, would impact their bean consumption. Most participants (68.9%) agreed that knowing more about how to prepare beans would encourage them to eat more of them, with 28.5% strongly agreeing and 40.4% somewhat agreeing. These responses suggest that knowledge about both the health benefits and preparation methods of beans is perceived as relevant to consumption frequency.

### 3.5. Knowledge About Beans

To explore participants’ confidence in knowledge about beans, participants provided self-reported confidence ratings regarding their knowledge of various aspects related to beans, as summarized in Table 2. Overall, the confidence levels were high across the different topics assessed. Many participants felt confident in their knowledge of the fibre content of beans, with 30.3% strongly agreeing and 46.4% somewhat agreeing. Similarly, 28.0% strongly agreed and 48.0% somewhat agreed that they were confident in their knowledge of the protein content. In terms of the health benefits of beans, 31.8% strongly agreed and 46.0% somewhat agreed. The highest level of confidence was reported for the knowledge that beans are naturally gluten-free, with 53.2% of respondents strongly agreeing and 32.0% somewhat agreeing. The lowest level of confidence was reported for knowledge of how to prepare beans, with 25.5% of respondents strongly agreeing and 31.2% somewhat agreeing.

### 3.6. Interest in Learning More About Beans

**Figure 4:**
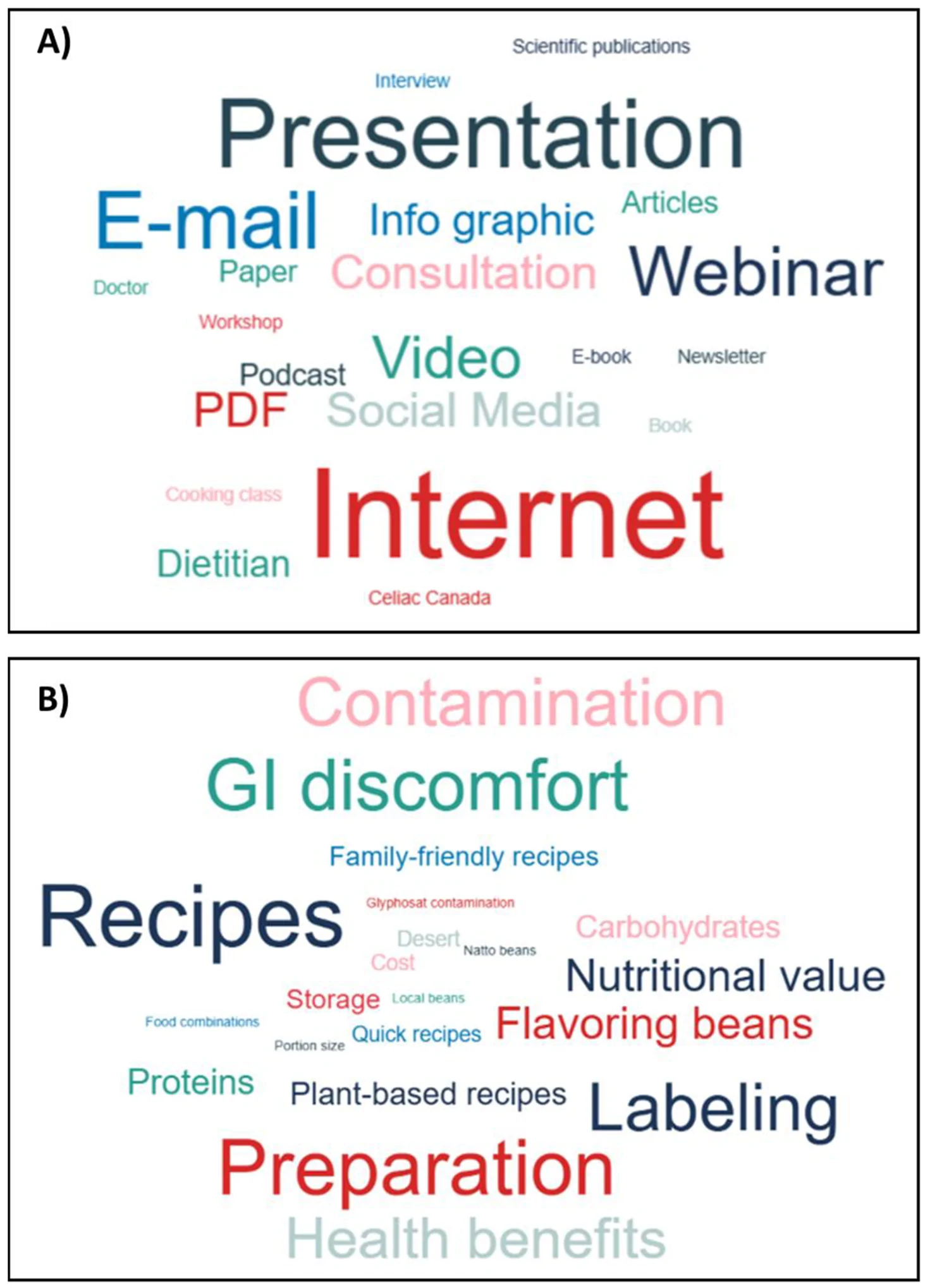
The word cloud is based on associations with an interest in beans. These associations are derived from all responses to the question: “Would you like to learn more about beans? If yes, what would you like to learn more about, and in which format would you like this information to be delivered?”. (**A**) Preferred formats for receiving information, (**B**) Topics participants are interested in learning more about. The data used for the word cloud is log-transformed.

To explore participants’ interest in beans, those with CD and NCGS were asked about their willingness to learn more about beans. An opportunity was also provided to specify which topics they would like to explore and how they would prefer to receive this information. Overall, 63.0% of participants agreed with the statement “I would be interested to learn more about beans,” while only 12.9% indicated they were not interested in further information. Preferred formats and topics for receiving information about beans are presented in Figure 4. These formats and topics were commonly identified by a large population of respondents (*n* = 306), including the internet (*n* = 98) as the most preferred format, followed by presentations (*n* = 33), emails (*n* = 19), webinars (*n* = 12), and videos (*n* = 8). Less frequently cited formats included consultations, infographics, podcasts, and cooking classes, reflecting interest in beans through a range of digital, written, and interactive formats. In terms of topic, the most frequently identified topics of interest were “Recipes” (*n* = 80), “Preparation” (*n* = 52), and “Gastrointestinal discomfort” (*n* = 38). Participants expressed particular interest in quick and family-friendly recipes and strategies to prevent or decrease gastrointestinal discomfort associated with bean intake. Additional topics of interest were “Health benefits” (*n* = 30), “Contamination” (*n* = 30) and “Nutritional value” (*n* = 18). Regarding contamination, participants were primarily interested in identifying beans and bean-derived products with low risk of gluten cross-contamination and sold without allergen labels indicating possible gluten traces.

### 3.7. Drivers of Bean Consumption

The overall bean consumption score ranged from 2 to 12, with a mean of 4.90 (SD = 1.44) and a median of 5 (interquartile range: 4–6). Scores were concentrated between 4 and 6, with 5 being the most frequent (*n* = 163, 30.9%). Only 24 participants (4.5%) had scores of 8 or higher (Figure S1). Two multivariable linear regression models were used to examine factors associated with overall bean consumption among participants with CD or NCGS. Model A included 389 participants, and Model B included 415 participants with complete data. Regression estimates and 95% confidence intervals were calculated using heteroskedasticity-consistent HC3 standard errors.

Model A included 389 participants and explained 25.4% of the variance in overall bean consumption scores (R^2^ = 0.254; adjusted R^2^ = 0.187). The motivators dietary preferences (B = 0.491, 95% CI: 0.149 to 0.833), cost (B = 0.425, 95% CI: 0.105 to 0.745), and sustainability (B = 0.556, 95% CI: 0.094 to 1.019) showed positive associations with bean consumption at the nominal significance level (*p* < 0.05). In contrast, the barriers gastrointestinal discomfort (B = −0.403, 95% CI: −0.701 to −0.104), limited cooking knowledge (B = −0.342, 95% CI: −0.657 to −0.027), and dislike of beans (B = −0.633, 95% CI: −1.247 to −0.020) showed negative nominal associations. The barrier family preferences showed a positive nominal association (B = 0.414, 95% CI: 0.001 to 0.827). However, none of the 20 individual motivator or barrier indicators remained statistically significant after Benjamini–Hochberg correction for multiple testing (all adjusted *p* ≥ 0.062). These findings were therefore interpreted as exploratory (Table S1).

Model B included 415 participants and explained 30.4% of the variance in overall bean consumption scores (R^2^ = 0.304; adjusted R^2^ = 0.265). Each additional reported motivator was associated with a 0.141-point higher consumption score (B = 0.141, 95% CI: 0.086 to 0.196; *p* < 0.001), whereas each additional reported barrier was associated with a 0.121-point lower score (B = −0.121, 95% CI: −0.218 to −0.024; *p* = 0.015), holding the other variables constant. Greater interest in learning more about beans was positively associated with bean consumption (B = 0.303, 95% CI: 0.176 to 0.431; *p* < 0.001), as was greater self-reported confidence in knowledge of bean preparation (B = 0.246, 95% CI: 0.146 to 0.345; *p* < 0.001). The remaining self-reported confidences in knowledge and the frequencies of animal- and plant-based-product consumption were not statistically significant at the nominal level (Table S2).

**Figure 5:**
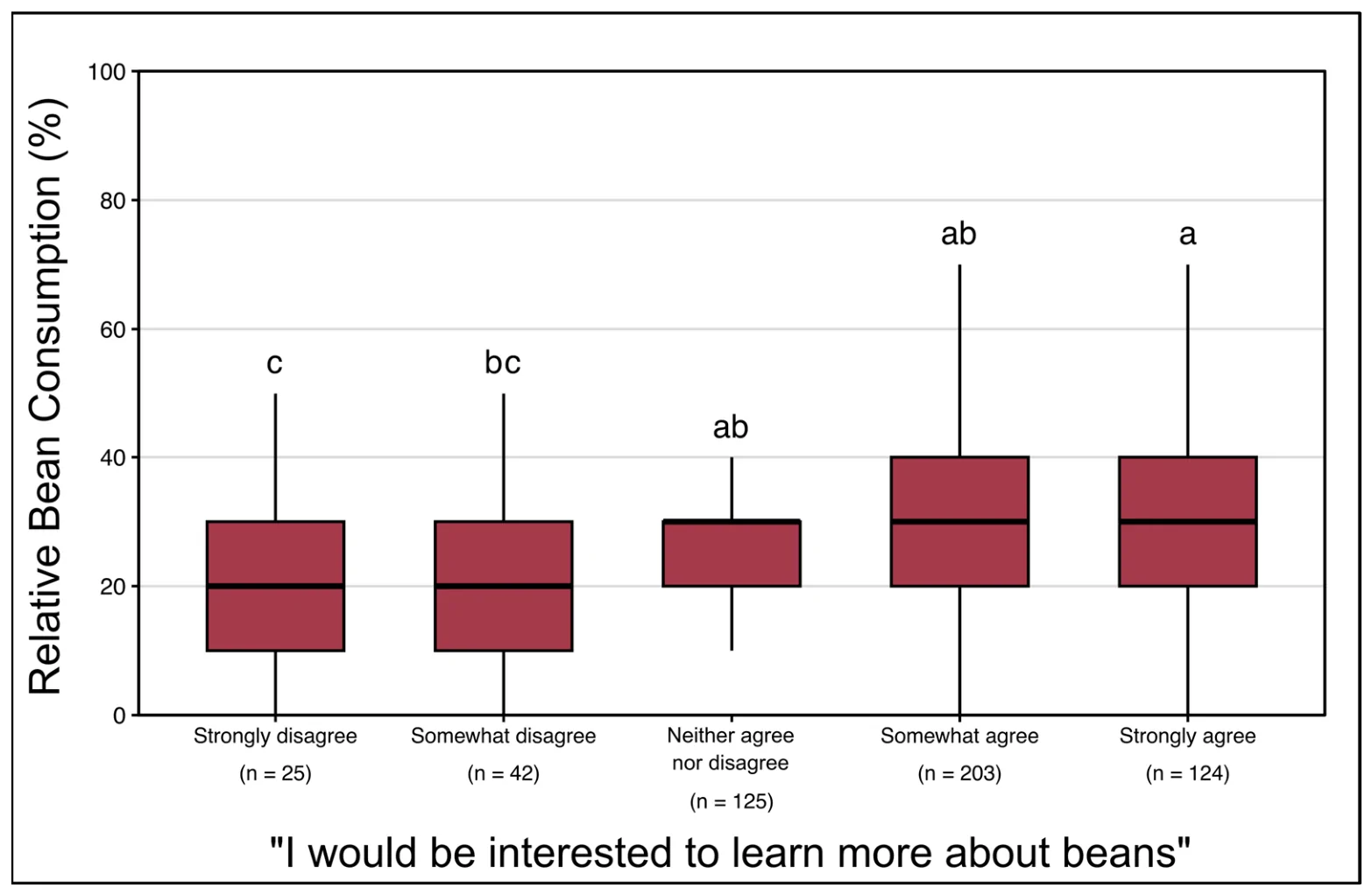
Relative bean consumption (%) among participants according to their level of agreement with the statement: “I would be interested in learning more about beans.” Relative bean consumption (%) was calculated as [(overall bean consumption frequency score − 2)/10] × 100. Boxplots show medians and interquartile ranges (IQR); whiskers extend to 1.5 × IQR, and outliers are not displayed. Differences were assessed using the Kruskal–Wallis test followed by Dunn’s multiple comparison test with Bonferroni correction. Different lowercase letters indicate significant differences between groups (*p* < 0.05).

Exploratory analyses were conducted to examine selected factors associated with bean consumption outside the multivariable linear regression models. Figure 5 presents relative bean consumption across levels of interest in learning more about beans. Consumption generally increased with greater reported interest. Participants who strongly or somewhat agreed that they were interested in learning more about beans had significantly higher relative bean consumption than those who somewhat or strongly disagreed. Participants who neither agreed nor disagreed also had significantly higher relative bean consumption than those who strongly disagreed, based on Dunn’s pairwise comparisons with Bonferroni correction following the Kruskal–Wallis test.

**Figure 6:**
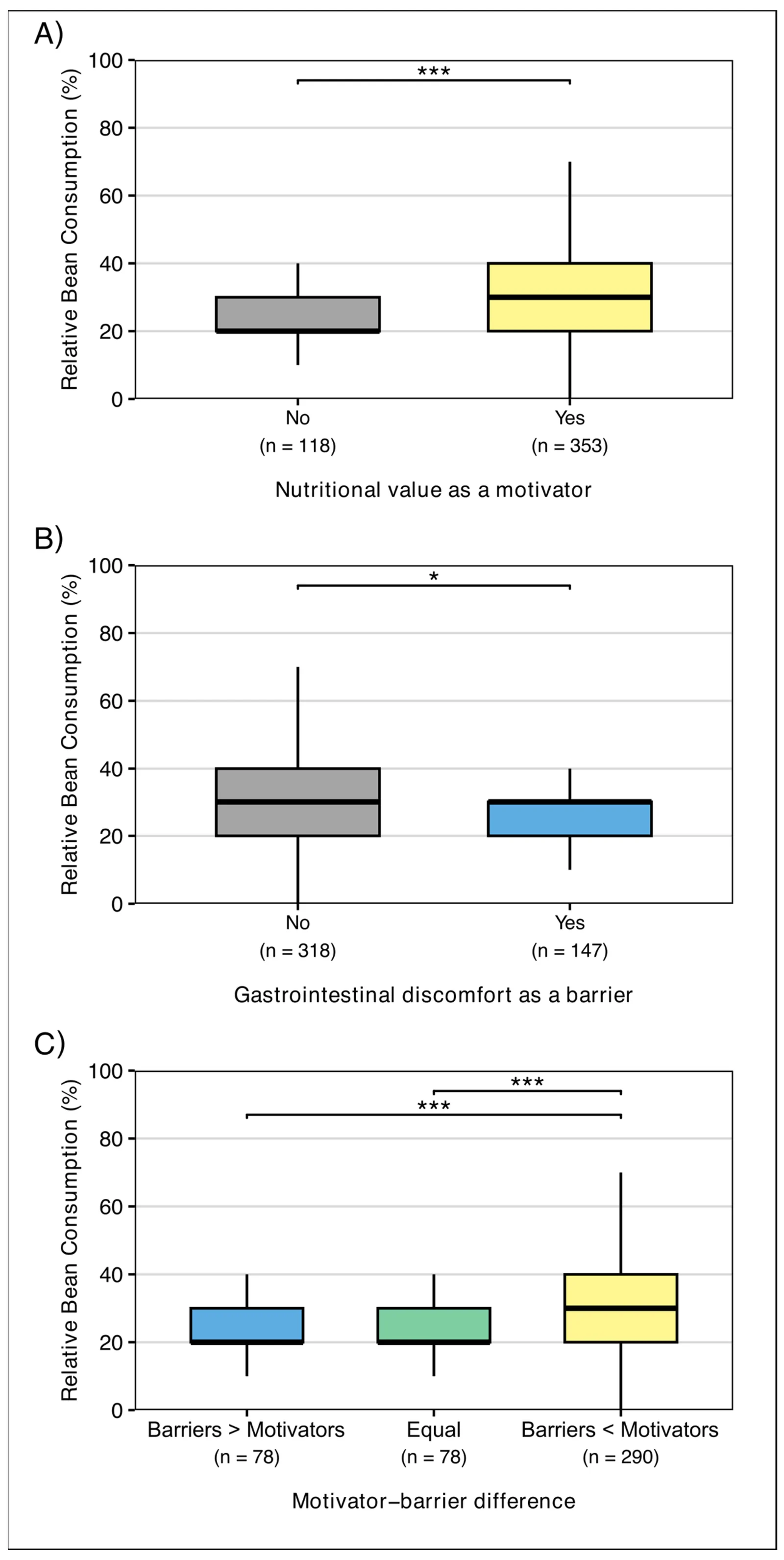
Relative bean consumption (%) according to (**A**) nutritional value as a motivator (“No” vs. “Yes”), (**B**) gastrointestinal discomfort as a barrier (“No” vs. “Yes”), and (**C**) the motivator-barrier difference. Relative bean consumption (%) was calculated as [(overall bean consumption frequency score − 2)/10] × 100. Boxplots show medians and interquartile ranges (IQR); whiskers extend to 1.5 × IQR, and outliers are not displayed. Differences were assessed using Mann–Whitney U tests (**A**,**B**) and Kruskal–Wallis followed by Bonferroni-adjusted Dunn tests (**C**). Significance: *p* < 0.05 (*), *p* < 0.001 (***).

The motivator nutritional value was examined as a motivator in relation to bean consumption (Figure 6A). Participants who identified nutritional value as a motivator (*n* = 353) had significantly higher relative bean consumption than those who did not (*n* = 118; *p* < 0.001). Similarly, gastrointestinal discomfort was examined as a barrier to bean consumption (Figure 6B). Participants who did not report gastrointestinal discomfort as a barrier (*n* = 318) had significantly higher relative bean consumption than those who did (*n* = 147; *p* = 0.045). Both comparisons were assessed using Mann–Whitney U tests. Figure 6C illustrates differences in relative bean consumption according to whether participants reported more motivators than barriers, equal numbers of motivators and barriers, or more barriers than motivators. Participants reporting more motivators than barriers (*n* = 315) had higher relative bean consumption than those reporting equal numbers (*n* = 116; *p* < 0.001) or more barriers than motivators (*n* = 97; *p* < 0.001). No significant difference was found between the groups reporting equal numbers and more barriers than motivators.

## 4. Discussion

This is one of the first studies to explore the knowledge and consumption of beans as part of the GFD among individuals with CD or NCGS in Canada.

### 4.1. Low Level of Bean Consumption

The overall frequency of bean consumption among participants was assessed based on the combined intake of dry and canned beans. Around 39.8% of participants reported a low consumption of beans. This is consistent with findings from previous research. For instance, older adults in Germany consume legumes on average only twice per month [22]. In general, dry beans were infrequently consumed compared to canned beans, with the majority (52.7%) reported consuming canned beans once a week or less. This difference can be attributed to convenience. Canned beans are ready to eat and require no preparation, whereas dry beans require significantly more time and planning [23]. Although dry beans are approximately two to three times less expensive than canned beans [24], their time-intensive preparation may hinder regular consumption, particularly among individuals with limited cooking skills or time.

### 4.2. High Interest in Beans and Its Impact on Consumption

The majority of participants (63.0%) expressed interest in learning more about beans, which may be explained by the ongoing dietary concerns experienced by individuals with CD and NCGS, including ensuring nutritional adequacy, avoiding cross-contamination, and managing social and dietary restrictions. Previous research has shown that these concerns are common in this population, with 43.7% of individuals with CD reporting challenges related to maintaining a healthy and balanced GFD [25]. In addition, beans may represent a practical and cost-effective alternative in the context of the limited availability and higher cost of gluten-free products [16].

Consistent with prior literature, participants indicated a preference for receiving information about beans primarily through the internet, followed by presentations and email [26]. However, the quality and reliability of online health information vary considerably. Therefore, disseminating accurate, evidence-based information through the digital platforms most frequently used by individuals with CD and NCGS is important.

The most frequently requested topics for further information included recipes, preparation methods, gastrointestinal tolerance, and concerns regarding gluten cross-contamination. Interest in recipes and preparation strategies may be driven by the limited variety of gluten-free options available in restaurants and retail settings, as well as a desire for greater dietary variety and enjoyment in eating [27]. In this context, beans may represent a valuable addition to the GFD by expanding food choices and improving dietary diversity. However, preparation of beans can present practical barriers. Providing clear, practical, and accessible preparation guidance may therefore facilitate greater incorporation of beans into the diets of individuals with CD or NCGS.

Furthermore, individuals with CD and NCGS frequently experience gastrointestinal symptoms, including diarrhea, steatorrhea, abdominal pain, and flatulence [5]. This may partly explain participants’ interest in gastrointestinal tolerance related to bean consumption. Beans are known to cause intestinal gas and bloating due to their high content of dietary fibre and resistant starches [28]. These findings highlight that gastrointestinal symptoms are a sensitive and relevant concern for individuals with CD and NCGS, underscoring the importance of providing clear and evidence-based information regarding the introduction and consumption of beans within a GFD. Cross-contamination was another key topic of interest among participants. Concerns may arise from the fact that beans and bean-based products are not always labelled as gluten-free and may be perceived as at risk of contamination. In Canada, the use of a gluten-free label is voluntary [11], meaning that naturally gluten-free foods such as beans may not carry this designation even when safe for consumption. However, cross-contamination is not uncommon in the food supply [29] and even naturally gluten-free foods may be perceived as risky if their production and handling conditions are not clearly communicated. Previous research indicates that gluten-free labelling plays an important role in supporting food choices among individuals with CD, with 54.7% reporting increased suspicion when products are not labelled gluten-free [30]. In addition, label attributes such as written claims and symbols can influence purchasing and selection behaviours [31]. This likely contributes to the strong interest observed in cross-contamination-related information among participants. To conclude, these findings suggest that greater interest in nutrition may be associated with healthier dietary choices, including increased incorporation of nutrient-dense, naturally gluten-free foods such as beans among individuals with CD or NCGS.

### 4.3. Confidence in Bean Knowledge and Its Impact on Consumption

Participants reported generally high confidence in their knowledge of beans. The highest level of confidence was observed for knowledge that beans are naturally gluten-free (85.2%). In contrast, confidence in bean preparation was notably lower, with only 56.7% of participants reporting confidence in this area. The high level of confidence in the nutritional attributes of beans may be partly explained by the relatively high educational attainment of the study population. However, this confidence did not extend to practical preparation skills. One possible explanation is the wide variety of bean types available, including kidney, black, pinto, and navy beans, each of which may require different preparation techniques [32]. This variability may contribute to uncertainty regarding appropriate preparation methods. These findings suggest that while participants are generally aware of the nutritional benefits of beans, a lack of confidence in preparation may represent a barrier to their consumption. Consequently, practical educational interventions, such as recipe resources, cooking demonstrations, or hands-on cooking classes, may help increase confidence and facilitate greater incorporation of beans into the GFD.

The findings of the present study are consistent with previous research demonstrating positive perceptions of the nutritional value of beans. For example, a Canadian food survey found that older adults recognized the health benefits associated with bean consumption [33]. Similarly, an Australian cross-sectional study reported that legumes were commonly associated with favourable nutritional attributes, including protein, fibre, and carbohydrate content, and were frequently perceived as suitable alternatives to meat [18]. Collectively, these findings suggest that awareness of the nutritional benefits of legumes is relatively widespread across diverse populations.

In addition, multivariable linear regression analysis revealed a positive association between self-reported confidence in knowledge of bean preparation and frequency of bean consumption. This suggests that participants who are more confident in their preparation skills tend to consume beans more often. Previous research demonstrating that higher levels of education and nutrition knowledge are associated with greater consumption of fruits, vegetables, and dietary fibre [34,35].

Overall, these findings underscore the potential role of education in promoting bean consumption among individuals with CD or NCGS. Increasing knowledge and confidence related to the nutritional value and preparation of beans may be associated with greater incorporation of this nutrient-dense, naturally gluten-free food into the diet. Educational resources that combine nutrition information with practical preparation guidance may be particularly effective in translating knowledge into dietary behaviour.

### 4.4. The Role of Perceptions in Bean Consumption

The most common reasons that motivated participants to eat beans were the nutritional value of beans, their use as a vegetarian alternative, and their affordability. Beans possess a high nutritional value due to their content of protein, essential amino acids, fibre, vitamins, and minerals. They are particularly rich in minerals, such as iron, zinc, and copper, as well as in vitamins, including folate, tocopherols, and thiamin [36]. In contrast, many gluten-free products are highly processed and high in fat, sugar, and sodium, and low in fibre and protein [37]. In addition, individuals with CD often have deficiencies in nutrients such as iron, zinc, vitamins B12 and D, and folate [38]. This finding aligns with previous research showing that nutritional value is a key motivator for consuming beans in other populations. For instance, a Canadian food survey reported that 93.8% of older adults consume beans due to their nutritional value [16].

Furthermore, beans are naturally gluten-free and served as a vegetarian option. They are a nutrient-rich alternative to meat and fish, offering high protein content and a favourable amino acid profile. Plant-based meat alternatives are often made from legume protein due to its affordability and functional processing properties [39]. In addition, the cultivation of beans is sustainable and has a low negative impact on the environment [40]. Participants may have been motivated to include beans in their diet due to their plant-based nature, gluten-free status, and environmental sustainability.

In addition, the price of food can be a major burden for those affected by CD or NCGS. According to the CCA, gluten-free products are between 150% and 500% more expensive than their gluten-containing counterparts. Consequently, about half of those affected by CD or NCGS report limited affordability of gluten-free foods [11]. In contrast to most gluten-free products, the retail price of beans in Canada is relatively low, ranging from CAD 1.34 to CAD 4.00 per kilogram [41]. This might explain why cost and affordability are motivating factors for consuming beans among individuals with CD and NCGS.

In contrast, the most common factors hindering participants from eating beans were the long cooking time, lack of habitual consumption and gastrointestinal discomfort associated with beans. In fact, dried beans, in particular, take a lot of time and effort to prepare. One way cooking time can be shortened is by soaking beans in advance [24] or using canned beans, which require minimal preparation.

Furthermore, beans not being part of a habit was another frequently mentioned barrier to consumption. However, only 0.4% of participants reported that they were unwilling to try new foods. In contrast to the study population, bean consumption is more prevalent in developing and low-income countries, where beans represent a dietary staple [42]. It is therefore not surprising that participants in this study do not consider beans a regular part of their diet.

Gastrointestinal discomfort was also frequently mentioned as a barrier to bean consumption. This is particularly relevant for individuals with CD or NCGS, who are commonly affected by gastrointestinal symptoms due to their disorder [5]. Moreover, beans may cause gastrointestinal discomfort because they contain oligosaccharides, such as raffinose and stachyose, that cannot be digested by human enzymes. Instead, these carbohydrates are fermented by gut bacteria, producing gases such as hydrogen, carbon dioxide, and methane. This process can lead to bloating and flatulence [17]. These findings are consistent with previous studies that identified gastrointestinal discomfort as a significant barrier to consuming beans [17,43].

In general, most participants (*n* = 328) reported more motivators than barriers to bean consumption. This suggests that participants generally have a favourable perception of beans.

The reported motivators and barriers to bean consumption were also reflected in participants’ actual eating behaviour. Exploratory comparisons showed that participants who identified nutritional value as a motivator reported significantly higher bean consumption compared to those who did not. Similarly, participants who did not report gastrointestinal discomfort as a barrier consumed beans more frequently than those who did. This relationship may reflect an association between perceived benefits and barriers, which are key factors shaping dietary behaviour. In addition, participants who reported more motivators than barriers consumed significantly more beans. Therefore, the overall difference between motivators and barriers was associated with bean consumption frequency. These findings underscore the importance of addressing both perceived benefits and barriers to effectively promote bean consumption, particularly in health-conscious populations such as individuals with CD or NCGS.

## 5. Strengths and Limitations

This study presents some strengths and limitations. A strength of this study includes the expert review and pretesting of the questionnaire before being implemented on the larger sample of participants. However, although these steps supported the questionnaire’s content and comprehensibility, the psychometric properties of the derived measures, including the bean-consumption score and its categorization, were not formally evaluated. Furthermore, a considerable sample size was used in this study. Although previous research has used questionnaires to investigate knowledge and attitudes toward legume or bean consumption, their sample sizes were notably smaller [16,17]. However, recruitment primarily through the CCA and academic networks may have resulted in a sample that is more health-conscious, knowledgeable about nutrition, and highly educated than the general population with CD or NCGS. Furthermore, both the recruitment materials and the questionnaire were distributed exclusively online, which may have excluded individuals without internet access or limited digital competence. This may disproportionately affect older individuals, people with lower socioeconomic status, and those who are less familiar with digital technology [44]. In addition, the sample is not representative of the broader Canadian population affected by CD or NCGS as these disorders occur across all ethnicities and genders. Although women are about 1.5 times more likely to be affected by CD [4], they were still notably overrepresented in this study. This trend has also been observed in previous research, where women made up around 85% of study populations [45,46]. Several methodological limitations should also be considered when interpreting the findings. The cross-sectional design does not allow causal relationships to be established or the temporal direction of observed associations to be determined. Furthermore, the diagnosis of CD or NCGS and other health-related information were self-reported and were not independently verified, potentially introducing misclassification. Residual confounding by unmeasured or inadequately measured factors cannot be excluded. The bean-consumption score was derived from reported consumption frequencies for the assessed food items and may not fully capture overall bean intake. In particular, the questionnaire did not include other bean-containing products, such as bean-based pasta or tortilla wraps, which may have resulted in an underestimation of bean consumption.

## 6. Conclusions

This study emphasizes the potential of beans in a GFD for individuals with CD or NCGS. While there is a high level of interest in beans and confidence in knowledge about them is high, actual consumption remains low. This highlights a clear opportunity to promote beans as a valuable dietary component, given their rich nutritional profile and their potential contribution to nutrient intake among individuals with CD or NCGS. The findings underscore the potential value of incorporating beans into the diets of affected individuals as one option to support a nutritionally balanced GFD. To this purpose, practical strategies should be developed and widely distributed. These strategies could include bean-based, gluten-free recipes and cooking classes, information on the differences between bean varieties, preparation tips, and guidance on possible gastrointestinal side effects. Organizations such as Celiac Canada, registered dietitians, and social media platforms can play a key role in providing this information and promoting the consumption of beans. Future research could focus on human intervention studies to assess the tolerability and long-term effects of bean consumption in CD and NCGS populations. In addition, more detailed nutritional assessments could further explore the role of beans as one of several valuable dietary options in a nutrient-rich GFD.

## Figures and Tables

**Table 1:** Demographic characteristics of the participants.

Demographic Characteristics	Individuals with CD or NCGS *n* (%) ^1^
Gender—Reported: 485, Unreported: 43	
Men	37 (7.6)
Women	443 (91.3)
Non-binary/third gender	3 (0.6)
Prefer not to disclose	1 (0.2)
Other gender identity	1 (0.2)
Age (years)—Reported: 481, Unreported: 47	
18–25	27 (5.6)
26–35	78 (16.2)
36–45	109 (22.7)
46–55	84 (17.5)
56–65	98 (20.4)
65 or older	85 (17.7)
Ethnicity—Reported: 500, Unreported: 28	
Indigenous	12 (2.4)
White/European	459 (91.4)
Black/Africa/Caribbean	3 (0.6)
Latin American	6 (1.2)
Southeast Asian	6 (1.2)
South Asian	9 (1.8)
Arab	1 (0.2)
Other	4 (0.8)
Education—Reported: 485, Unreported: 43	
No diploma	2 (0.4)
Secondary diploma or equivalent	53 (10.9)
College certificate or diploma	108 (22.3)
Bachelor’s degree	160 (33.0)
University diploma above bachelor’s degree	150 (30.9)
Other	12 (2.5)
Household income (Canadian $)— Reported: 424, Unreported: 104	
<25,000	20 (4.7)
$25,000–$49,999.99	36 (8.5)
$50,000–$74,999.99	63 (14.9)
$75,000–$99,999.99	78 (18.4)
$100,000+	227 (53.5)
Province—Reported: 484, Unreported: 44	
Alberta	63 (13.0)
British Columbia	73 (15.1)
Manitoba	20 (4.1)
New Brunswick	8 (1.7)
Newfoundland and Labrador	12 (2.5)
Northwest Territories	0 (0.0)
Nova Scotia	21 (4.3)
Nunavut	0 (0.0)
Ontario	243 (50.2)
Prince Edward Island	5 (1.0)
Quebec	19 (3.9)
Saskatchewan	15 (3.1)
Outside of Canada	5 (1.0)

^1^ Values are reported as *n* (%), where *n* indicates the number of participants and % indicates the percentage of valid responses of each variable.

**Table 2:** Self-reported confidence in bean-related knowledge among participants with celiac disease or non-celiac gluten sensitivity.

	Number of Participants	Strongly Disagree (%)	Somewhat Disagree (%)	Neither Agree nor Disagree (%)	Somewhat Agree (%)	Strongly Agree (%)
I feel confident about my knowledge on the fibre contained in beans.	519	6.0	7.1	10.2	46.4	30.3
I feel confident about my knowledge on the protein contained in beans.	517	4.6	7.2	12.2	48.0	28.0
I feel confident about my knowledge on the health benefits of beans.	515	4.1	7.2	10.9	46.0	31.8
I feel confident about my knowledge that beans are natural gluten-free food options.	515	3.9	5.0	5.8	32.0	53.2
I feel confident about my knowledge on the way to prepare beans.	510	8.4	17.3	17.6	31.2	25.5

## Data Availability

Due to confidentiality reasons, data may only be shared upon request from the corresponding author.

## Supplementary Materials

The following supporting information can be downloaded at: https://www.mdpi.com/article/10.3390/nu18213553/s1, Table S1: Multivariable linear regression of overall bean consumption scores using individual motivators and barriers (Model A); Table S2: Multivariable linear regression of overall bean consumption scores using motivator and barrier counts, self-reported confidence in bean-related knowledge, interest in learning more about beans and dietary habits (Model B); Figure S1: Distribution of overall bean consumption scores among study participants (*n* = 528). The overall bean consumption score was calculated by summing self-reported dry- and canned-bean consumption frequency scores, each coded from 1 (never) to 6 (daily), yielding a theoretical range of 2–12. Higher scores indicate more frequent bean consumption. Bars represent the number of participants at each score.
