# Supplementary material for: Exploring Knowledge and Consumption of Beans Among Adults with Gluten-Related Disorders—A Cross Sectional Study in Canada
Source: Nutrients. 2026 Oct 28;18(21):3553. doi: 10.3390/nu18213553 (PMC13765056; doi:10.3390/nu18213553)
Supplement: Supplementary file 1 [file nutrients-18-03553-s001.zip › nutrients-4572300-supplementary.pdf]

Supplementary Material

Exploring Knowledge and Consumption of Beans Among Adults with Gluten-Related Disorders—A Cross Sectional Study in Canada

Alicia Stahl [1], Alyssa Chapleau [1], Sana Tasawar [2], Safa Shahid [1], Niels J. Maywald [3], Nanette Stroebele-Benschop [4], Iris Joye [5] and Dalia El Khoury [1,*]

[1] Department of Family Relations and Applied Nutrition, University of Guelph, Guelph, ON N1G 2W1, Canada
[2] Department of Human Health Sciences, University of Guelph, Guelph, ON N1G 2W1, Canada
[3] Department of Nutritional Crop Physiology, University of Hohenheim, Fruwirthstraße 20, 70599 Stuttgart, Germany
[4] Department of Applied Nutritional Psychology, University of Hohenheim, Fruwirthstraße 12, 70599 Stuttgart, Germany
[5] Department of Food Science, University of Guelph, Guelph, ON N1G 2W1, Canada
* Correspondence: delkhour@uoguelph.ca

Table S1. Multivariable linear regression of overall bean consumption scores using individual motivators and barriers (Model A).

Predictor	B	SE	95% CI	p-value	BH-adjusted p
Intercept	5.151	0.763	3.650 to 6.652	<0.001	—
Motivator: Nutritional value	-0.087	0.180	-0.441 to 0.267	0.630	0.807
Motivator: Vegetarian option	0.137	0.156	-0.170 to 0.444	0.381	0.693
Motivator: Dietary preferences	0.491	0.174	0.149 to 0.833	0.005	0.062
Motivator: Taste	-0.089	0.163	-0.409 to 0.231	0.584	0.807
Motivator: Cost	0.425	0.163	0.105 to 0.745	0.009	0.062
Motivator: Ease of preparation	-0.070	0.177	-0.419 to 0.279	0.694	0.807
Motivator: Versatility	0.159	0.176	-0.186 to 0.504	0.365	0.693
Motivator: Long shelf life	0.117	0.148	-0.175 to 0.409	0.430	0.717
Motivator: Sustainability	0.556	0.235	0.094 to 1.019	0.019	0.093
Motivator: Canadian origin	0.066	0.175	-0.279 to 0.410	0.709	0.807
Motivator: Cultural reasons	-0.041	0.330	-0.690 to 0.609	0.902	0.902
Barrier: Gastrointestinal discomfort	-0.403	0.152	-0.701 to -0.104	0.008	0.062
Barrier: Not part of a habit	-0.278	0.147	-0.568 to 0.011	0.059	0.148

Predictor	B	SE	95% CI	p-value	BH-adjusted p
Barrier: Cooking time	0.042	0.142	-0.237 to 0.320	0.769	0.810
Barrier: Limited cooking knowledge	-0.342	0.160	-0.657 to -0.027	0.034	0.134
Barrier: Taste	-0.162	0.265	-0.684 to 0.361	0.543	0.807
Barrier: Dislike of beans	-0.633	0.312	-1.247 to -0.020	0.043	0.142
Barrier: Traditional eating patterns	-0.204	0.172	-0.542 to 0.134	0.237	0.526
Barrier: Family preferences	0.414	0.210	0.001 to 0.827	0.049	0.142
Barrier: Unspecific reason	-0.144	0.413	-0.956 to 0.667	0.727	0.807
Age	0.000	0.005	-0.009 to 0.010	0.938	—
Gender: Non-binary / third gender	0.396	1.475	-2.505 to 3.297	0.789	—
Gender: Women	-0.184	0.341	-0.854 to 0.486	0.589	—
Education: College certificate or diploma	0.451	0.201	0.056 to 0.847	0.025	—
Education: Other	-0.201	0.351	-0.891 to 0.489	0.568	—
Education: Secondary school diploma	0.048	0.254	-0.451 to 0.547	0.851	—
Education: University diploma above bachelor's	0.082	0.157	-0.227 to 0.390	0.603	—
Household income: $100,000+	-0.355	0.469	-1.277 to 0.568	0.450	—
Household income: $25,000–$49,999	-0.242	0.554	-1.331 to 0.847	0.663	—
Household income: $50,000–$74,999	-0.373	0.475	-1.308 to 0.562	0.433	—
Household income: $75,000–$99,999	-0.445	0.492	-1.413 to 0.523	0.367	—
Diagnosis: Non-celiac gluten sensitivity	-0.314	0.266	-0.836 to 0.209	0.239	—

Note. B = unstandardized regression coefficient; SE = HC3 heteroskedasticity-robust standard error; CI = 95% confidence interval. The outcome was the overall bean consumption score. The model included all 20 individual motivator and barrier indicators, adjusted for age, gender, education, annual household income, and diagnosis. Reference categories were Man, bachelor's degree, annual household income below $25,000, and celiac disease. BH-adjusted p-values were calculated using the Benjamini–Hochberg procedure across the 20 individual motivator and barrier tests only. N = 389; R^2 = 0.254; adjusted R^2 = 0.187.

Table S2. Multivariable linear regression of overall bean consumption scores using motivator and barrier counts, self-reported confidence in bean-related knowledge, interest in learning more about beans and dietary habits (Model B).

Predictor	B	SE	95% CI	p-value
Intercept	3.991	1.319	1.405 to 6.578	0.003

Predictor	B	SE	95% CI	p-value
Number of motivators	0.141	0.028	0.086 to 0.196	<0.001
Number of barriers	-0.121	0.050	-0.218 to -0.024	0.015
Interest in learning more about beans	0.303	0.065	0.176 to 0.431	<0.001
Knowledge of bean fiber	0.016	0.084	-0.148 to 0.180	0.849
Knowledge of bean protein	0.071	0.081	-0.087 to 0.229	0.380
Knowledge of health benefits	0.009	0.090	-0.167 to 0.185	0.922
Knowledge of gluten-free suitability	-0.056	0.084	-0.220 to 0.109	0.508
Knowledge of bean preparation	0.246	0.051	0.146 to 0.345	<0.001
Animal-product consumption frequency	-0.162	0.100	-0.357 to 0.034	0.106
Plant-product consumption frequency	0.022	0.115	-0.203 to 0.248	0.847
Age (years)	-0.004	0.004	-0.013 to 0.004	0.307
Gender: Non-binary / third gender	0.106	1.477	-2.789 to 3.001	0.943
Gender: Women	-0.330	0.330	-0.976 to 0.316	0.317
Education: College certificate or diploma	0.382	0.180	0.030 to 0.735	0.034
Education: High school diploma or less	0.005	0.225	-0.435 to 0.445	0.984
Education: Other	-0.419	0.367	-1.138 to 0.300	0.254
Education: University diploma above bachelor's	0.053	0.148	-0.238 to 0.344	0.721
Household income: $100,000+	-0.193	0.479	-1.132 to 0.746	0.687
Household income: $25,000–$49,999	-0.436	0.557	-1.527 to 0.655	0.434
Household income: $50,000–$74,999	-0.359	0.487	-1.313 to 0.596	0.462
Household income: $75,000–$99,999	-0.395	0.492	-1.358 to 0.568	0.422
Diagnosis: Non-celiac gluten sensitivity	-0.378	0.265	-0.896 to 0.141	0.154

Model B: n = 415; R^2 = 0.304; adjusted R^2 = 0.265. B = unstandardized coefficient; SE = HC3 robust standard error; CI = confidence interval. Reference categories: Man, bachelor's degree, annual household income <$25,000, and celiac disease.

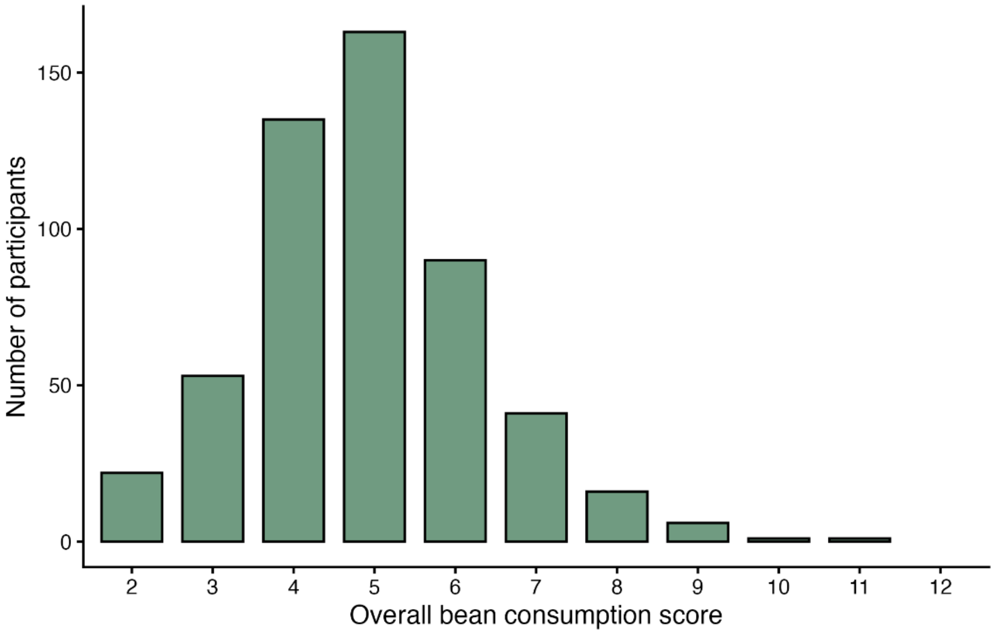

Figure S1. Distribution of overall bean consumption scores among study participants (n = 528). The overall bean consumption score was calculated by summing self-reported dry- and canned-bean consumption frequency scores, each coded from 1 (never) to 6 (daily), yielding a theoretical range of 2–12. Higher scores indicate more frequent bean consumption. Bars represent the number of participants at each score.
